# Myxoid glioneuronal tumor of the septum pellucidum with concurrent dual *PDGFRA* and *FGFR3* gene mutations: a case report and literature review

**DOI:** 10.3389/fonc.2026.1903894

**Published:** 2026-09-01

**Authors:** Meng Wang, Lingyan Wang, Lili Zhang, Jianwei Zhang, Yonghui Yang, Li Zhang

**Affiliations:** Department of Pathology, Children’s Hospital of Hebei Province, Hebei Provincial Clinical Research Center for Child Health and Disease, Shijiazhuang, Hebei, China

**Keywords:** dysembryoplastic neuroepithelial tumor, immunohistochemistry, myxoid glioneuronal tumor, next-generation sequencing, septum pellucidum

## Abstract

Myxoid glioneuronal tumor (MGNT), a rare neuroepithelial neoplasm newly recognized in the 2021 World Health Organization (WHO) Classification of Tumors of the Central Nervous System (5th edition), is associated with platelet‐derived growth factor receptor α (*PDGFRA)* gene alterations. While MGNT typically exhibits indolent histopathological features and a favorable clinical course, rare cases of intraventricular dissemination and leptomeningeal metastasis have been documented. We report a case of a 10-year-old female who presented with headache and was found to have an MGNT in the septum pellucidum. Gross total resection was achieved, and histopathological examination confirmed the diagnosis. Targeted next-generation sequencing (NGS) revealed concurrent pathogenic variants in *PDGFRA* and fibroblast growth factor receptor 3 (*FGFR3).* To contextualize this finding, we performed a systematic review of published MGNT literature to synthesize its clinicopathological, immunohistochemical, and molecular features. Our analysis indicates that integrating immunohistochemistry with molecular profiling—especially NGS—is essential for precise diagnosis and risk stratification. This case broadens the known molecular spectrum of MGNT and implies *FGFR3* as a potential therapeutic target, warranting further investigation of FGFR-directed inhibitors in selected patients.

## 1 Introduction

MGNT was originally classified as a dysembryoplastic neuroepithelial tumor (DNET). The WHO subsequently redefined it as MGNT in its 2021 classification of central nervous system (CNS) tumors, based on its distinct anatomical location, histological morphology, and molecular pathology (1). Fewer than one hundred cases have been reported globally, so its clinicopathological features remain incompletely characterized due to its rarity (2). MGNT is classified as WHO Grade 1, with a predilection for septum pellucidum. It follows an indolent clinical course, and while cerebrospinal fluid (CSF) dissemination has been suggested, distant metastasis is exceedingly rare (3).

At the molecular level, most cases of MGNT harbor a highly specific genetic alteration: a dinucleotide substitution at codon 385 of the *PDGFRA* oncogene, which substitutes lysine with leucine or isoleucine (4). This mutation serves as a critical molecular hallmark for differential diagnosis. The clinical significance of concomitant mutations in MGNT, however, remains insufficiently characterized. Furthermore, although cases in atypical locations like the lateral ventricles and midbrain tectum are increasingly documented (3, 5), more detailed radiological and pathological data are needed to enhance preoperative diagnostic accuracy.

Herein, we report a case of MGNT originating in the septum pellucidum in a patient presenting with headache. Through histomorphological examination and NGS, we not only confirmed the classic *PDGFRA* mutation but also identified a concurrent *FGFR3* mutation. The aim of this study is to explore the clinicopathological features and complex molecular genetic background of MGNT through an in-depth analysis of this typical yet rare case, thereby providing new insights for the precise diagnosis of this tumor entity.

## 2 Case presentation

**Figure 1:**
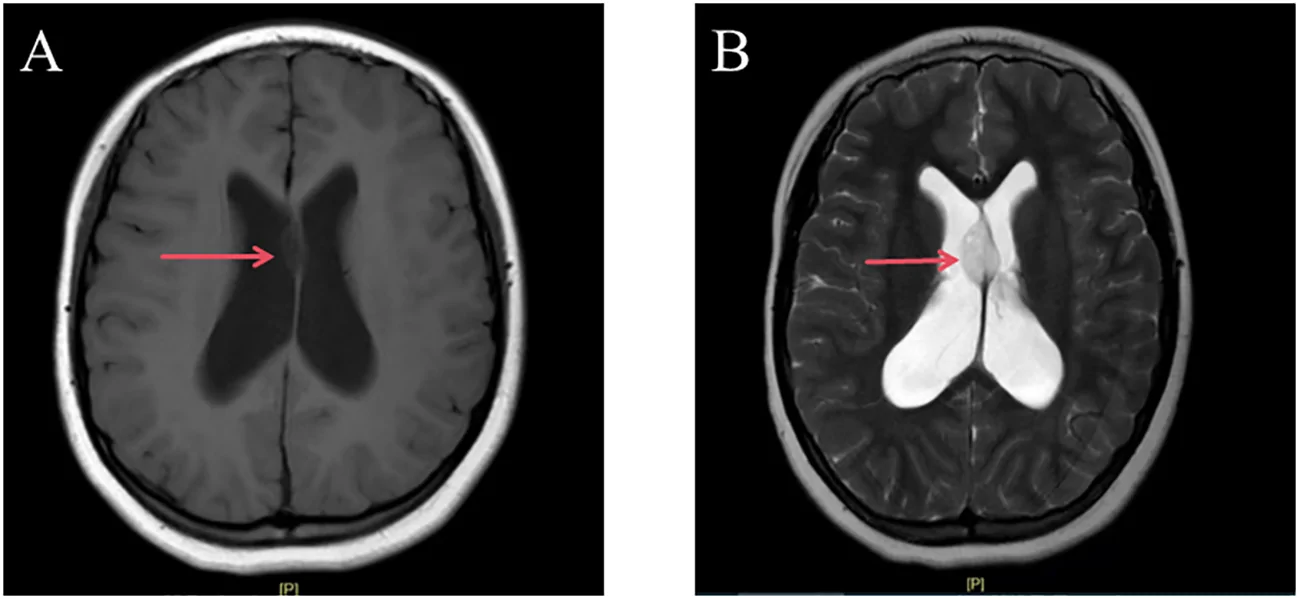
MRI showing bilateral hydrocephalus and a mass at the foramen of Monro. **(A)** Axial T1-weighted image demonstrating dilated lateral ventricles and a hypointense mass (arrow) at the foramen of Monro. **(B)** Axial T2-weighted image showing the same mass (arrow) appearing hyperintense, with associated ventriculomegaly.

**Figure 2:**
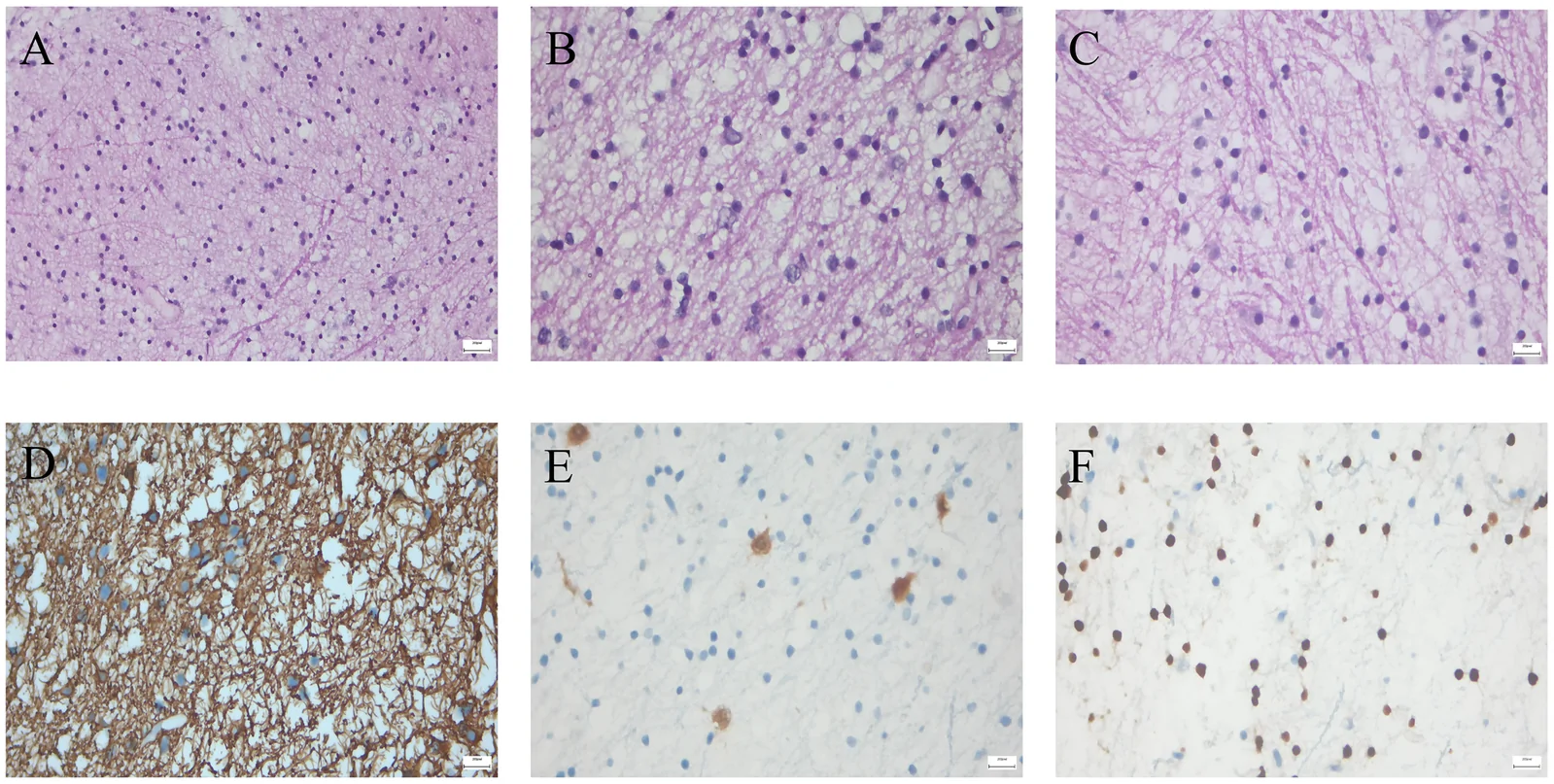
Hematoxylin and eosin (H&E) and immunohistochemical staining. The background is mucoid with scattered small neurons [**(A)** ×200; **(B)** ×400]. Tumor cells are small and granular [**(C)** ×400]. Immunohistochemistry shows varying degrees of positivity for GFAP, NeuN, and Olig-2 [**(D–F)**, ×400].

A 10-year-old female patient was admitted due to “headache without apparent cause”. The pain was localized to the orbital region and accompanied by non-projectile vomiting. The patient was conscious, with no cranial deformity. Pupils were equal and round (2.5 mm), with prompt light reflex and no sunset sign. The neck was supple, with negative meningeal irritation signs. Limb muscle tone was slightly increased. Vital signs were stable, and body temperature was normal. Laboratory findings were unremarkable. Brain Magnetic Resonance Imaging (MRI) revealed bilateral hydrocephalus and a fusiform midline mass in the region of the foramen of Monro, which was T1 hypointense, T2 hyperintense with partially suppressed signal on Fluid-Attenuated Inversion Recovery (FLAIR) and a peripheral hyperintense rim (**Figure 1**). A tumor resection was performed 8 days after admission. Following incision of the corpus callosum and septum pellucidum, a 2.5 cm × 2 cm × 2.0 cm grayish-white, soft, solid mass was exposed and piecemeal resected under microscopy. Microscopically, the tumor exhibited a typical oligodendroglial-like morphology. The tumor cells displayed regular, round-to-oval nuclei with vacuolated, clear cytoplasm. The cytological features were bland, and no mitotic figures were identified. The tumor background was characterized by a prominent myxoid matrix with scattered neuronal elements (**Figure 2**). Immunohistochemistry (IHC) showed diffuse positivity of the lesional cells for Vimentin, Glial Fibrillary Acidic Protein (GFAP), Oligodendrocyte Lineage Transcription Factor 2 (Olig-2) and S-100 Protein (S-100), with intact Alpha Thalassemia/Mental Retardation Syndrome X-Linked (ATRX) nuclear expression. Consistent with the mixed glial-neuronal differentiation spectrum of this myxoid glioneuronal tumor, Neurofilament (NF) and Synaptophysin (SYN) showed focal immunoreactivity limited to the neuronal component, while the oligodendroglial-like tumor cells were negative for both markers. Neuron-specific nuclear protein (NeuN) staining was focally positive. The tumor showed no immunoreactivity for Spalt Like Transcription Factor 4 (SALL4), Epithelial Membrane Antigen (EMA), and p53. The Antigen KI-67 (Ki-67) proliferation index was low (~3%), and BRAF V600E was negative. NGS analysis of the submitted specimen demonstrated pathogenic missense mutations in *FGFR3* (NM_022965.4): p.V441L (c.1321G>T) and p.C433S (c.1299T>A), and in *PDGFRA* (NM_006206.6): p.K385M (c.1154A>T). Variant Allele Frequency (VAF) was not reliably determined by this assay. Based on the histology, immunophenotype, and molecular genetics, a diagnosis of myxoid glioneuronal tumor was established. At the 3-month postoperative follow-up, the patient occasionally reported headache and nausea, with negative findings on neurological examination. Postoperative contrast-enhanced brain MRI revealed focal thickening and enhancement of the meninges in the right frontal region, with no definite radiological evidence of tumor recurrence detected in the remaining intracranial compartments. By the 6-month postoperative follow-up, the extent of the previously noted thickened and enhanced meninges had significantly regressed compared with the prior imaging study, and no other abnormal intracranial findings were observed.

## 3 Literature review

This study reviewed 28 cases of MGNT from 11 published studies (**Table 1**). Although various anatomical locations have been reported in the literature, the septum pellucidum (32%, 9/28) remains the predilection site for this tumor. Demographic analysis revealed that MGNT occurs in patients ranging from children to middle-aged adults, with a slight male predominance (16 males vs. 12 females) (2, 5–12). Clinically, most patients presented as asymptomatic (4%, 1/28) or with headaches (54%, 15/28) at initial diagnosis, while others exhibited neurological symptoms, including cognitive impairment, ataxia, and loss of consciousness (43%, 12/28) (9). CSF dissemination (4%, 1/28) was observed in a subset of cases. Regarding prognosis, the vast majority of patients demonstrated a favorable outcome, with no evidence of recurrence or metastasis during follow-up. NGS analysis revealed that the predominant mutation in this cohort was *PDGFRA* p.K385L/I, detected in 75% of patients (21/28). The remaining alterations included *PDGFRA* p.E362I363insW (4%, 1/28) and a previously unreported *Notch receptor 1(NOTCH1)* mutation (4%, 1/28). The molecular profile of the remaining five cases was not specified in the original reports (3).

**Table 1:** Cases of MGNT reported in the literature (n=28).

First author/s	Cases, n	Primary sites	Age,years	Sex	Greatest tumor diameter	Clinical symptoms	IHC		Gene mutation	Diffusion
							Positive markers	Ki-67 (%)		
Ahmed Gilani (6)	1	Temporal lobe	10	Female	27mm	Epileptic	NeuN+	N/A	PDGFRA p. K385L	N/A
Jiayi Chu et al. (7)	1	Septum pellucidum	N/A	Male	2mm	Headache,Diz-zy	Olig-2, SOX-10	1~3	N/A	N/A
Shoko Wakisaka et al. (10)	1	Septum pellucidum	19	Female	25mm	Headache	Olig-2	N/A	PDGFRAp.K385L	N/A
							SOX-10			
Eeuardo De Oliveira Narvaez et al. (9)	3	Left subcallosal gyrus,Subcortical white matter and left septal area	43.3 (mean)	Male (2), Female (1)	N/A	Headache,dis-turbance of behavior and Loss of Consciousnes-s	N/A	N/A	N/A	None
C. Caporalini et al. (13)	6	Corpus callosum and periventricular white matter of the lateral ventricle (4) and Septum pellucidum	10.2 (mean)	Male (4), Female (2)	N/A	Headaches, Emesis, Seizures and behavioral disturbances	Olig-2, GFAP,NeuN and NF	1~3	PDGFRAp.K385L/I	None
B.K.Kleinschmidt-DeMasters etal (3)	1	Midbrain tectum	41	Female	N/A	Head and neck pain	Olig-2	N/A	PDGFRAp.K385L, NOTCH1	Cerebrospinal fluid
Calixto-Hope G.Lucas et al. (5)	8	Septum pellucidum (4), callosum (3) and Periventricular white matter (1)	23.6 (mean)	Male (4), Female (4)	N/A	Headache,Co-gnitive disorder,ataxi-a and Asymptomati-c state	Olig-2, SOX-10	1~4	PDGFRAp.K385L/I	N/A
Serena Salzano et al. (8)	1	Periventricula-r white matter	29	Male	18	Headache	GFAP, Olig-2, SYN	1	PDGFRAp.K385L	N/A
Kadir Oktay et al. (2)	1	Septum pellucidum	10	Male	N/A	Headache,Epi-lepsy	Olig-2, SYN	1	N/A	None
Alena Stasenko et al. (11)	1	Septum pellucidum	30+	Female	22	Asymptomati-c state	GFAP,NeuN,SYN	1~5	PDGFRAp.K385L	None
Zejun Duan et al. (12)	4	Interventricular foramen	27	Female	N/A	Headache and Visual impairment	S-100, GFAP, SYN	1-4	PDGFRAp.K385L/p. E362I363insW	None

N/A, no relevant information available; SOX-10, SRY-related HMG-box 10.

Given the rarity of pediatric MGNT in patients under 18 years of age, we conducted a retrospective analysis of the clinicopathological features of 6 reported cases, integrating data from existing literature. The cohort demonstrated an equal gender distribution (male: female = 1:1). Clinical manifestations were heterogeneous, ranging from incidental detection to symptoms of increased intracranial pressure (headache), seizures, and ataxia (2, 6, 13). Anatomically, the lesions were predominantly located in the midline and periventricular regions, specifically involving septum pellucidum, corpus callosum, and periventricular area. Regarding prognosis, one case exhibited disease progression post-resection, while the remaining cases remained recurrence-free during follow-up.

## 4 Discussion

MGNT is a rare CNS tumor, formally established in 2018 by Solomon et al. The authors described a neoplasm of the septum pellucidum exhibiting histological similarities to DNET and pilocytic astrocytoma (PA). Crucially, molecular analysis identified a dinucleotide substitution at *PDGFRA* codon p.k385, defining this entity as MGNT (14). Our case presents a tumor in the classic septum pellucidum location. Histopathological examination revealed oligodendroglia-like cells within a mucoid matrix, and immunohistochemical staining showed co-expression of GFAP, Olig-2. These findings align with the established MGNT phenotype. This case, characterized by its specific anatomy, histology, and *PDGFRA* mutation, reaffirms the diagnostic precision of the current WHO classification. It is worth noting that, in contrast to the widely documented *PDGFRA* p.K385L/I classic mutation, the *PDGFRA* mutation site detected in this case is P.K385M, which is a new variant with no previous research reports. This newly added mutation type also provides new clinical sample support for the molecular characteristics of this tumor subtype in the WHO classification system.

According to previously published radiological studies, the typical imaging manifestations of this tumor on Computed Tomography (CT) are low-density cystic or pseudocystic changes. On MRI, these classically described lesions show mixed hypo-to hyperintense signal on T1-weighted imaging (T1WI), markedly hyperintense signal on T2-weighted imaging (T2WI), and a characteristic pattern of partially suppressed central signal with a peripheral hyperintense rim on FLAIR sequences. These well-established reference imaging features are consistent with the radiological findings observed in our own case cohort (15).

The present case is distinguished by a unique *PDGFRA* p.K385M and *FGFR3* co-mutation signature identified via NGS. This is particularly noteworthy, as co-occurring *FGFR3* mutations have not been previously described in MGNT, which is typically driven by solitary *PDGFRA* p.K385L/I mutations. This distinct genetic profile implies an alternative pathogenic pathway. *PDGFRA*, a ubiquitously expressed *Receptor Tyrosine Kinase (RTK)*, drives cell growth and differentiation through ligand-activated signaling cascades. Its gain-of-function mutations are a key driver in multiple mesenchymal neoplasms, most notably Gastrointestinal stromal tumors (GIST) (16, 17). *FGFR3*, another *RTK*, is similarly crucial for growth, differentiation, and angiogenesis, and serves as a common oncogenic driver in urothelial carcinoma, and endometrial cancer, with emerging evidence implicating it in glioblastoma (18–21). We hypothesize a sequential model in which a *PDGFRA* mutation acts as an initiating event, followed by an *FGFR3* alteration that may serve as a cooperating lesion. While *FGFR3* alterations are known to activate *Mitogen-Activated Protein Kinase (MAPK)/Phosphoinositide 3-Kinase (PI3K)* pathways in other tumor contexts, the functional consequences of this specific mutational combination remain to be experimentally validated. Notably, this case exhibited no clinical evidence of tumor recurrence, metastasis, or elevated proliferative activity (Ki-67 index and mitotic count within normal ranges). Therefore, we do not interpret these findings as refuting the central role of *PDGFRA* in MGNT; rather, they expand the molecular spectrum of MGNT and highlight the possibility of additional genetic events. Whether this dual-mutational status defines a distinct molecular subgroup with altered biological behavior requires investigation in larger cohorts.

Although MGNT is classified as a WHO Grade 1 tumor and typically exhibits bland histological features (e.g., low mitotic count, low Ki-67 index), scattered reports have described rare cases of CSF dissemination or local recurrence, while the tumor still retains its intrinsically indolent biological properties. In the present case, postoperative follow-up MRI revealed dural thickening and enhancement in the right frontal region. Notably, molecular profiling identified an *FGFR3* mutation, which has been implicated in extracellular matrix remodeling and cell migration in other neoplasms. The transient meningeal enhancement, which spontaneously regressed on follow-up, likely represented postoperative inflammatory changes rather than tumor dissemination. Nevertheless, long-term surveillance remains prudent given the rarity of this entity.

Tumors in the septum pellucidum must be differentiated from PA, ependymoma, and DNET. PAs typically arise in the cerebellum and optic chiasm and are histologically characterized by dense bipolar pilocytic cells containing Rosenthal fibers, loose mucoid areas, and eosinophilic granular bodies. Immunohistochemically, tumor cells show strong expression of GFAP, Olig-2, and S100, while NeuN expression is typically absent. Genetically, the *KIAA1549: BRAF* gene fusion is a common feature (22). Ependymomas preferentially occur in the lateral ventricles and spinal cord. They are composed of round-to-oval nuclei with characteristic “salt-and-pepper” chromatin and rare mitotic figures. These tumors express EMA, GFAP, and S-100 but are negative for Olig-2. DNETs predominantly affect the cerebral cortex, most commonly the temporal lobe. Although their histological appearance is similar to that of the present case, they rarely occur in the septum pellucidum and lack *PDGFRA* mutations (23).

While surgery is currently the standard of care, the identification of *FGFR3* mutations in this entity provides a strong rationale for exploring targeted therapies. This approach holds particular promise for managing recurrence, dissemination, or cases where complete resection is not feasible due to tumor location.

## 5 Conclusion

We report a case of pediatric myxoid glioneuronal tumor (MGNT) in the septum pellucidum harboring co-mutations in *FGFR3* and *PDGFRA*. This finding not only expands the known genetic spectrum of MGNT and deepens our understanding of its underlying molecular heterogeneity, but also suggests that specific mutational combinations may be correlated with distinct clinical progression patterns of the tumor. Clinically, for such rare tumors, a “histo-molecular-radiological” integrated diagnostic and therapeutic model should be established. While aiming for gross total resection, clinicians must remain vigilant for the risk of dissemination and actively explore targeted therapeutic strategies against *RTK* pathway.

## Data Availability

The original contributions presented in the study are included in the article/supplementary material. Further inquiries can be directed to the corresponding author.
